# Supplementary material for: PIRT the TRP Channel Regulating Protein Binds Calmodulin and Cholesterol-Like Ligands
Source: Biomolecules. 2020 Mar 21;10(3):478. doi: 10.3390/biom10030478 (PMC7175203; doi:10.3390/biom10030478)
Supplement: Supplementary file 1 [file biomolecules-10-00478-s001.pdf]

Supplementary Materials

PIRT the TRP Channel Regulating Protein Binds Calmodulin and Cholesterol-like Ligands

Nicholas J. Sisco[1-4†], Dustin D. Luu[1-4†], Minjoo Kim[1-4], and Wade D. Van Horn[1-4,*]

[1] The School of Molecular Sciences, Arizona State University, Tempe, AZ 85287
[2] The Virginia G. Piper Biodesign Center for Personalized Diagnostics, Biodesign Institute, Arizona State University, Tempe, Arizona.
* Correspondence: wade.van.horn@asu.edu; Tel.: (480) 965-8322; Fax: (480) 965-2747

† These authors contributed equally to this work.

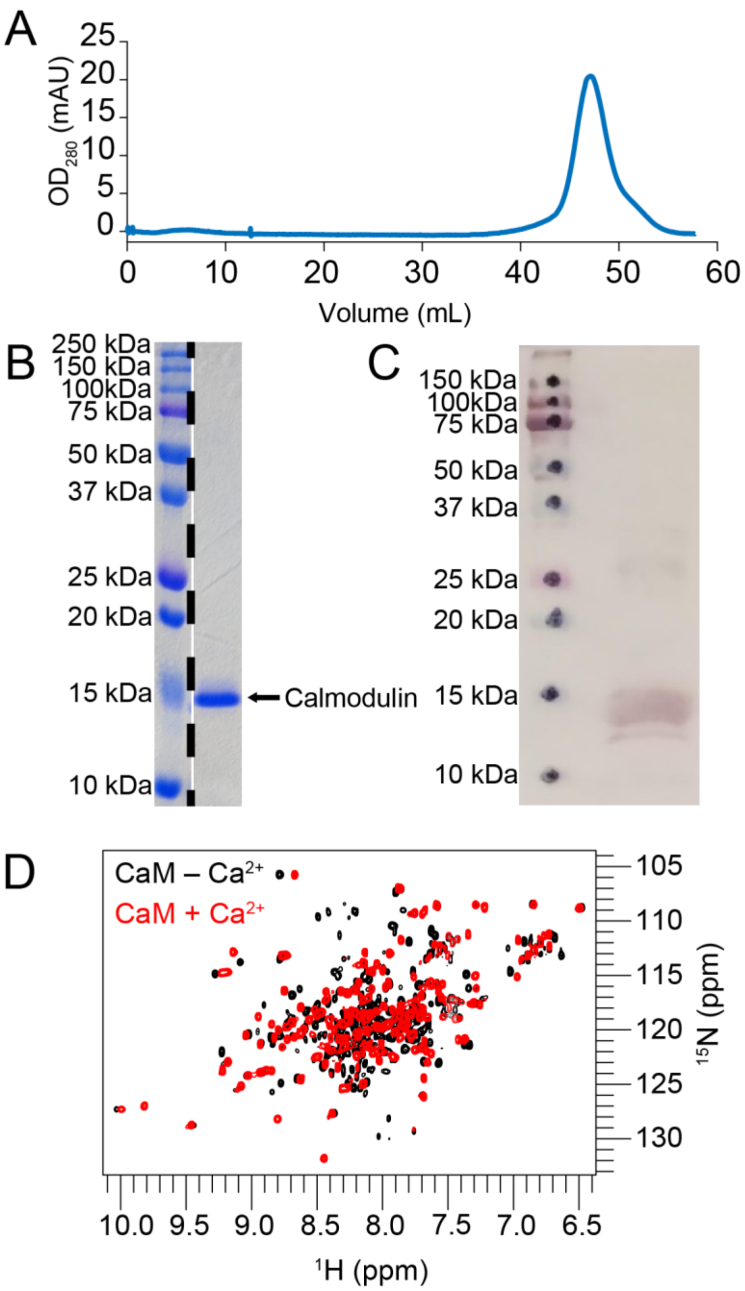

Figure S1: Characterization of Purified Calmodulin. (**A**) Purification of calmodulin with Ni-NTA and size exclusion chromatography (**B**) Purity was assessed with SDS-PAGE and (**C**) Identification with Western Blot of B. (**D**) Overlay of HSQC NMR spectra of ^{15}N-labeld apo (Ca^{2+}-free, black) and holo (Ca^{2+}-bound, red) calmodulin recorded at 25 °C.

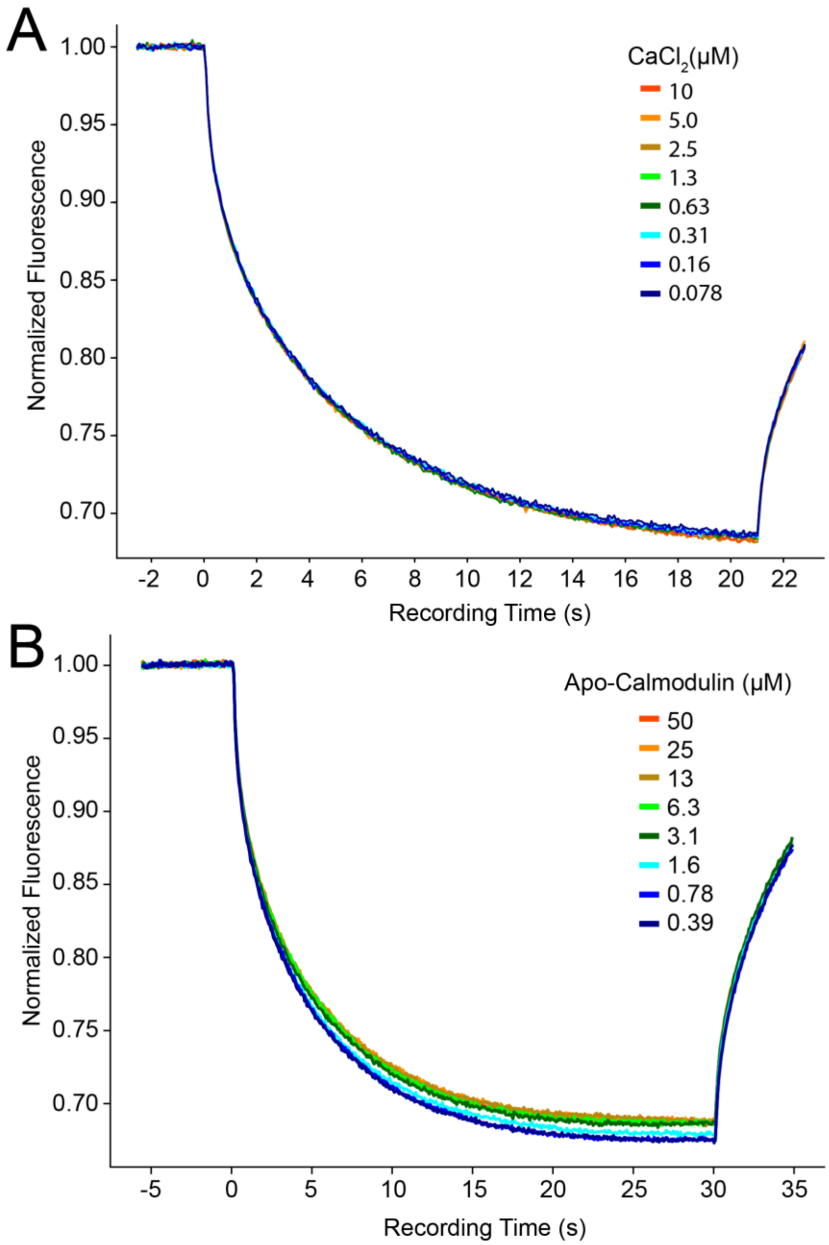

Figure S2: Representative PIRT microscale thermophoresis trace data. Non–binding was seen with varying concertation of (**A**) $CaCl_2$ with no coherent changes (Fig. S3A) in fluorescence. On the other hand, varying the concentration of (**B**) apo-calmodulin showed changes in fluoresce indicative of specific interactions (Fig. 2A) . The fluorescence was normalized to 1 from the initial relative fluorescence.

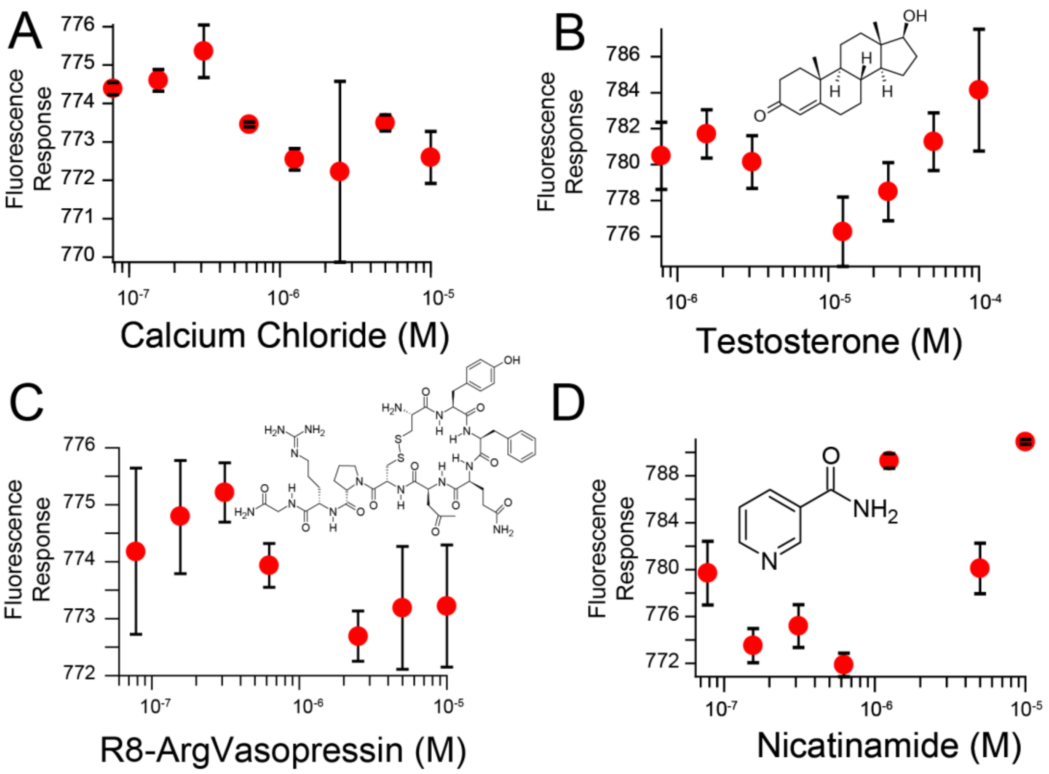

Figure S3: PIRT Ligand-independent thermophoresis. (**A**) Calcium chloride, (**B**) testosterone. (**C**) R8-arginine vasopressin and (**D**) niacinamide. There are small amounts of thermophoresis across the listed molecules; however, none of these fit to a binding isotherm.

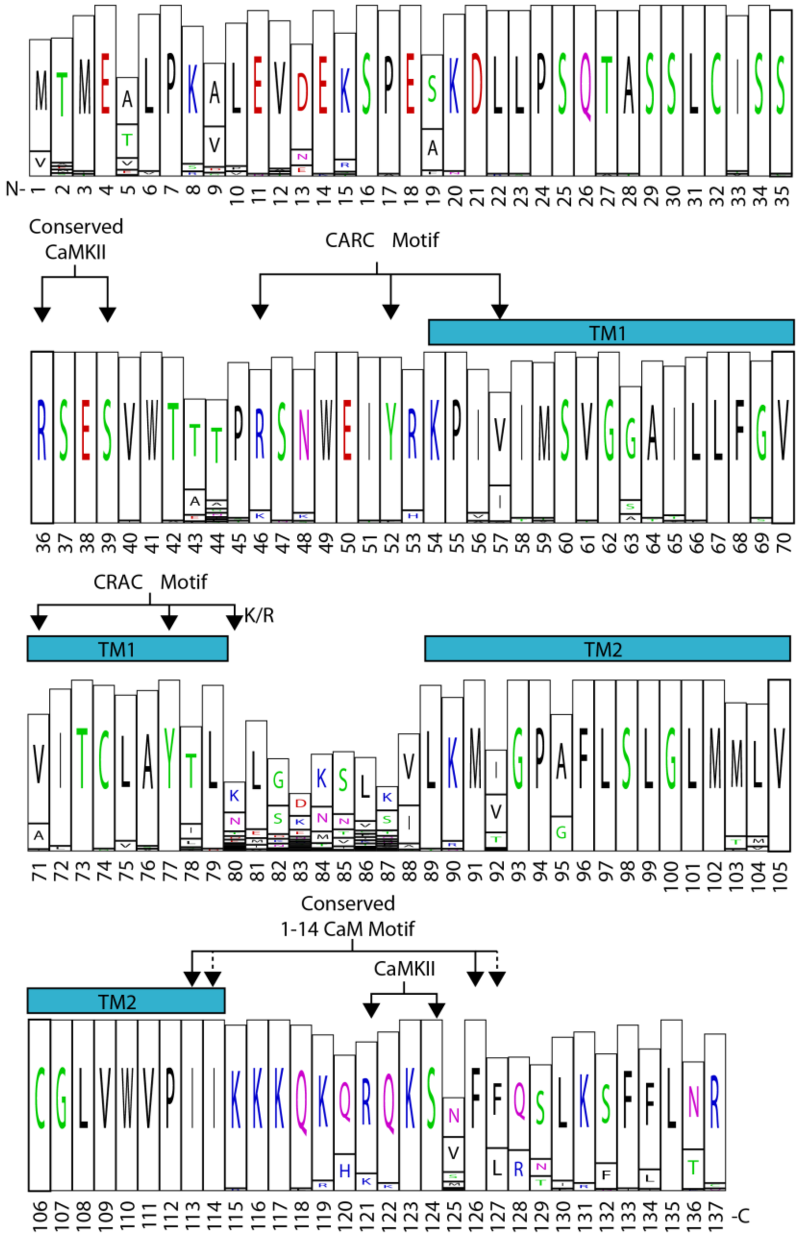

Figure S4: PIRT conservation across mammalian species. A web logo from multiple sequence alignment across mammalian species of PIRT [1]. Binding motifs for calmodulin, cholesterol, and calmodulin-dependent kinase II are show here as conserved across 59 sequences of proteins named as phosphoinositide-interacting proteins. The two transmembrane regions shown in blue-shade as TM1 and TM2, respectively. Arrows point to key residues in the motifs: for CaMKII (R-X-X-S/T), CRAC/CARC (R/K-X_{1-5}-Y/F- X_{1-5}-L/V), and 1-14 calmodulin motif ([FILVW]-X_{12}-[FILVW]). The height of the boxes stands for conservation across species with a taller box as more conservation. The 1-14 CaM binding motif is 100% conserved for solid arrows and 100% conserved for dotted arrows with the F replaced by L at the 14 position, which is still a key residue. CRAC and CARC domains show less conservation across the entire alignment, with the CRAC domain found outside of the TM1; however, we highlight it here to show the conservation. In human PIRT, the CARC domain is located at positions Ile72-Tyr77-Lys80. The N-terminal CaMKII phosphorylation motif is 100% conserved at position Arg36-Ser39 but is less conserved in the C-terminal motif.

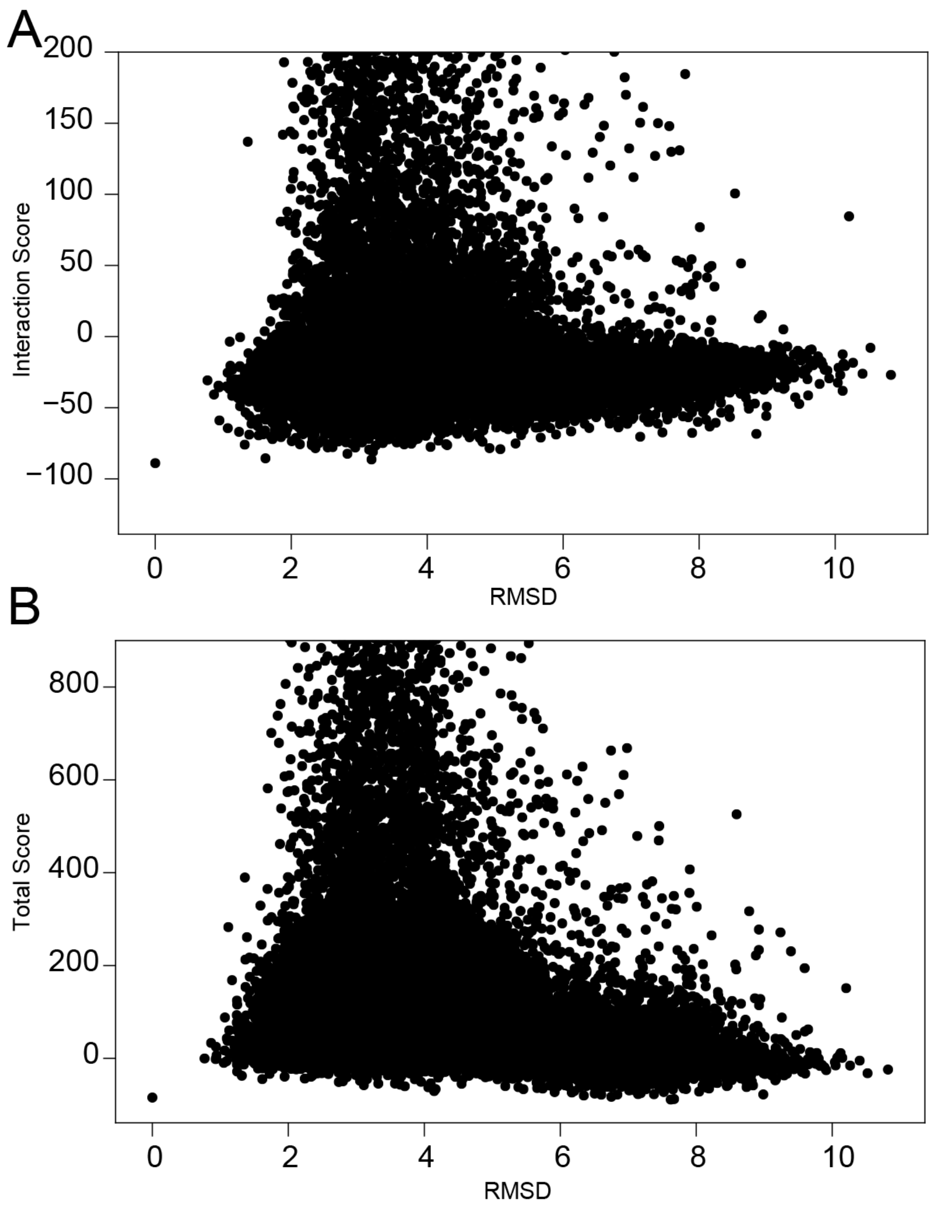

Figure S5: Unguided Rosetta ab initio flexible peptide docking. Docking shows good convergence for 80000 decoys of PIRT docking into calmodulin. (**A**) The interaction score is the difference between the scores of the individual chains scored independently during a docking run, and usually a smaller number suggests better docking interface. (**B**) The total score is the weight score from the output decoys, and a lower score represents lower energy and therefore more favorable. These results together show an energy funnel signifying convergence, with a broad range of RMSD showing a large conformation space was sampled.

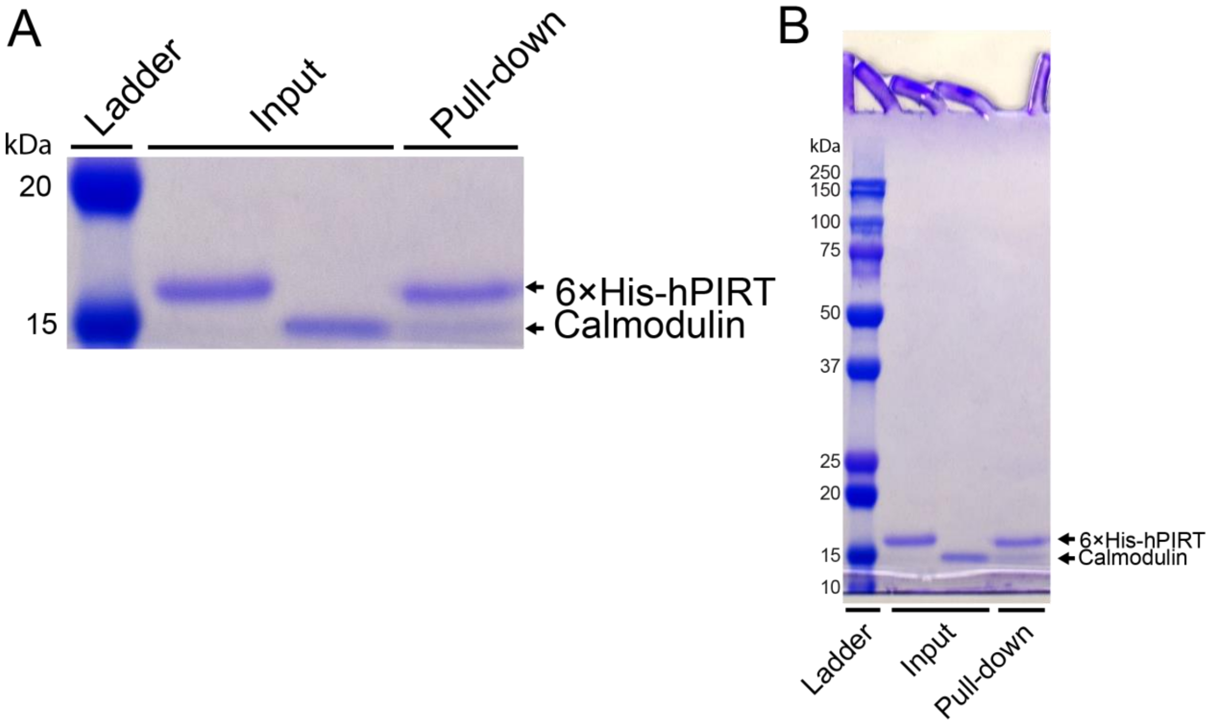

Figure S6. PIRT and calmodulin pull-down. (**A**) Coomassie-stained gel. His-tagged PIRT was mixed with cleaved calmodulin before binding to Ni-NTA resin. The bound complex was coeluted suggesting direct binding. (**B**) The SDS PAGE gel used for the cropped image shown in panel A.

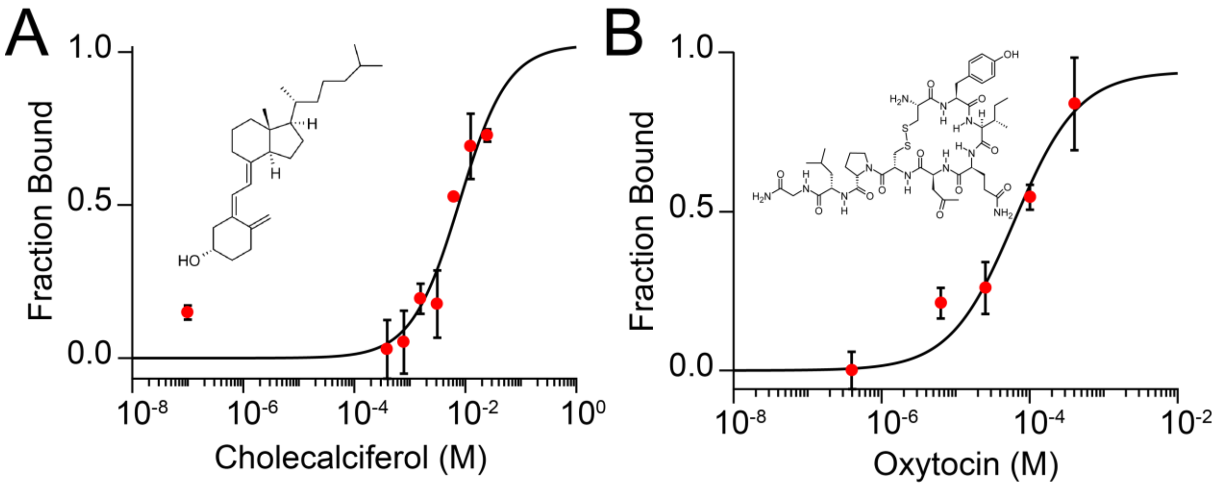

Figure S7: PIRT ligand-dependent thermophoresis. Thermophoresis of (**A**) cholecalcifereol (**B**) water soluble oxytocin. The K_d are shown in Table 1. The hydrophobic ligands became insoluble in the MST buffer conditions above the concentrations used.

References

1. Crooks, G.E. WebLogo: A Sequence Logo Generator. *Genome Res.* **2004**, *14*, 1188-1190, doi:10.1101/gr.849004.
